# Anti-MRSA and Biological Activities of Propolis Concentrations Loaded to Chitosan Nanoemulsion for Pharmaceutics Applications

**DOI:** 10.3390/pharmaceutics15102386

**Published:** 2023-09-26

**Authors:** Khaloud Mohammed Alarjani, Hany Mohamed Yehia, Ahmed Noah Badr, Hatem Salma Ali, Abdulrahman Hamad Al-Masoud, Sarah Mubark Alhaqbani, Shahad Ahmed Alkhatib, Ahmed Moustafa Rady

**Affiliations:** 1 Department of Botany and Microbiology, College of Science, King Saud University, P.O. Box. 2455, Riyadh 11451, Saudi Arabiasalhaqbani@ksu.edu.sa (S.M.A.); ahabdo@ksu.edu.sa (A.M.R.); 2 Food Science and Nutrition Department, College of Food and Agricultural Sciences, King Saud University, P.O. Box. 2460, Riyadh 11451, Saudi Arabia; hanyehia@ksu.edu.sa; 3 Food Toxicology and Contaminants Department, National Research Centre, Dokki, Giza 12622, Egypt; 4 Food Technology Department, National Research Centre, Dokki, Giza 12622, Egypt; hatem.owyean1@gmail.com

**Keywords:** antibacterial, anti-mycotoxigenic, anti-MRSA, propolis extract loading, phenolic fraction, nanoemulsion, chitosan

## Abstract

Propolis is a naturally occurring substance with beneficial properties; bees produce it from various plant sources, and it is an anti-inflammatory and therapeutic resinous substance. This study aimed to enhance the biological features of propolis extract by loading it onto active film. Firstly, extraction was performed using three solvent systems, and their total phenolic, flavonoid, and antioxidant activity was measured. Propolis ethanol extract (EEP) was evaluated for phenolic fraction content and then chosen to prepare a chitosan-loaded emulsion with several concentrations. The antibacterial, anti-mycotic, and anti-mycotoxigenic properties of the extract and nanoemulsion were assessed. PPE’s cytotoxicity and nanoemulsion were evaluated using brine shrimp and cell line assays. Results indicate higher phenolic (322.57 ± 4.28 mg GAE/g DW), flavonoid (257.64 ± 5.27 mg QE/g DW), and antioxidant activity of the EEP. The phenolic fraction is distinguished by 18 phenolic acids with high *p*-hydroxybenzoic content (171.75 ± 1.64 µg/g) and 12 flavonoid compounds with high pinocembrin and quercetin content (695.91 ± 1.76 and 532.35 ± 1.88 µg/g, respectively). Phenolic acid derivatives (3,4-Dihydroxybenzaldehyde, 3,4-Dihydroxyphenol acetate, and di-methoxy cinnamic) are also found. Concentrations of 50, 100, 150, and 200 ng EEP loaded on chitosan nanoemulsion reflect significant antibacterial activity against pathogenic bacteria, particularly methicillin-resistant *Staphylococcus aureus* (MRSA) and toxigenic fungi, particularly *Fusarium*. Among the four EEP-loaded concentrations, the nanoemulsion with 150 ng showed outstanding features. Using a simulated medium, 150 and 200 ng of EEP-loaded chitosan nanoemulsion concentrations can stop zearalenone production in *Fusarium* media with complete fungi inhibition. Also, it reduced aflatoxins production in *Aspergillus* media, with fungal inhibition (up to 47.18%). These results recommended the EEP-chitosan application for pharmaceutics and medical use as a comprehensive wound healing agent.

## 1. Introduction

Bees create a natural substance known as propolis, recently utilized in alternative medicine [1]. This resinous combination generally comes from plant sources (tree buds and sap flows). Propolis is mainly created to fortify the hive’s structure, lock openings, and prevent microbial contamination (bacteria, fungi, and pathogens) [2]. The methicillin-resistant *Staphylococcus aureus* (MRSA) strain is considered one of the great issues that can occur due to bacterial contamination. People have historically utilized propolis for various motives, including its possible health advantages. It comprises multiple components, such as flavonoid, phenolic, and other organic molecules [3]. Propolis is antibacterial against bacteria, viruses, and fungi, which might help inhibit pathogen development and promote wound healing; also, propolis anti-inflammatory effects and the potential to decrease inflammation in various illnesses have been investigated [1].

Methicillin-resistant *Staphylococcus aureus* (MRSA) is a pathogenic strain of harmful bacteria, also known as MRSA [4]. This microbe strain resists several pharmaceuticals, such as methicillin and other beta-lactam antibiotics [5,6]. The resistance of the MRSA infections poses significant challenges in their treatment, leading to a multitude of complications in both healthcare facilities and the wider population [7,8]. MRSA is resistant to numerous routinely used antibiotics, limiting treatment choices [9]. This results in more severe and protracted infections. MRSA is a frequent cause of hospital-acquired infections. MRSA may spread via intimate human contact or shared things such as towels and sports equipment [9]. These illnesses may result from skin abscesses, cellulitis, and other soft tissue infections [10]. MRSA may be discovered on the skin or nasal passages without causing symptoms. These carriers have the potential to transfer the pathogen to others accidentally. Some community-acquired MRSA strains are more virulent and may cause more severe illness [11,12]. Some antibiotics, such as vancomycin or daptomycin, may be beneficial against MRSA; however, these drugs may have adverse effects [13]. MRSA infections may be more severe and possibly fatal compared to non-resistant *Staphylococcus aureus* infections. Skin and soft tissue infections, pneumonia, bloodstream infections, and surgical site infections are all possible outcomes [14]. Another issue is that MRSA resistance genes might spread to other bacteria [15], possibly resulting in additional drug-resistant infections. This adds to the broader issue of antibiotic resistance, making it more challenging to treat diverse illnesses.

Propolis contains antioxidant substances that may help protect cells from oxidative damage produced by free radicals [16]. Phenolic compounds are phytochemicals known for their antioxidant and anti-inflammatory properties. The most common phenolic compounds in propolis extract are flavonoids, phenolic acids, and their derivatives. The propolis antioxidants’ activity varies by numerous factors, including their kind and uniqueness [17]. Propolis also possesses immunomodulatory properties, which may aid in regulating and maintaining the immune system. Pathogens may cause various infectious diseases in humans, from minor infections to life-threatening disorders. Pathogens with specific characteristics can generate outbreaks and epidemics [18]. Antibiotic overuse and abuse have led to the emergence of antibiotic-resistant bacteria, which may withstand the effects of medicines and make treatment more difficult. Antibiotic resistance is a worldwide health problem that may result in more prolonged illnesses, higher healthcare expenses, and fewer treatment alternatives [19]. These factors redirected the global interest to search for natural substances such as propolis.

Mycotoxin-producing fungi, commonly known as toxigenic fungi, threaten human health. Mycotoxins may infect food, other items, and buildings [20]. Long-term mycotoxin exposure or ingestion of large amounts may cause adverse health consequences. However, another fungal infection that may lead to several symptoms is Candida. Overgrowth of naturally occurring *Candida* species, most often *Candida albicans*, leads to candidiasis, sometimes a yeast infection [21]. White, creamy spots are a telltale sign of oral candidiasis, a common illness. However, using active compounds like propolis by scavengers can reduce the hazards of microorganism contamination [18]. *Candida* infections may increase immune system weakness, increase antibiotics or corticosteroid response, and cause certain medical issues [21]. Recently, some applications were recorded to enhance the antimicrobial and antifungal impacts of natural constituents’ antioxidant activity [22,23]. These applications are included in microemulsion and nanoemulsion, providing more activity and efficient properties. 

Nanoemulsion is a colloidal system of tiny particles of one liquid that are spread out in liquids that do not mix. Nanoemulsions and nanoparticle application are novel techniques that have biological interaction with several infection causes and reduces their severity [24]. Nanoemulsions received much attention in various areas, like medicine, food, makeup, and health applications [25,26]. Green synthesis of nanoparticles is considered a more safe technique used in the preparation [27]. Polymers like polyvinyl alcohol (PVA), chitosan, and carboxymethyl cellulose (CMC) are frequently used to create nanoemulsions because they emulsify and stabilize [28]. The CMC is a water-soluble cellulose derivative commonly used as a thickening agent, stabilizer, and emulsifier. Again, chitosan can form a gel-like structure at low pH, increasing bioavailability, viscosity, and stability. Generally, these materials can build a protective barrier around the active component being delivered, avoiding coalescence and ensuring the stability of the nanoemulsion. Because of its biocompatibility and non-toxicity, this polymer is appropriate for various applications, including medicines and therapeutic utilization. 

It is important to note that while propolis has shown promising potential, scientific research on its effectiveness and specific health benefits is still ongoing. The present investigation objective was to study the characteristics of Korean propolis (raw, nanoemulsion, and nanoparticle). Antimicrobial and antifungal properties were also assessed, particularly antibacterial and antibiofilm activities against pathogenic bacteria (particularly against MRSA-strain) and toxin-producing fungi. 

## 2. Materials and Methods

### 2.1. Chemicals and Materials 

Korean propolis powder (Princeherb^®^, Republic of Korea) was purchased from the Raydel Korea Mart, Gangnam-daero, Seocho-gu, Seoul, Republic of Korea. For the extraction and application processes, the powder was ground to near micronized granules (20 mesh) and instantly dried (40 ± 2 °C) in a hot-air oven (ED 56 Oven, Binder GmbH, Tuttlingen, Germany) until dried completely. Chitosan powder (low Mw: 100,000 to 120,000 Da; deacetylate degree: 75–85%). 

Microbial media of yeast extract sucrose (YES), nutrient agar (NA), Mueller–Hinton (MUH), synthetic nutrient broth (SNB), and Czapk-Dox agar (CZA) were utilized for the evaluation of antibacterial and antifungal activities. Each media was prepared according to the manufacturing methodology, and the application condition was adjusted according to the applied assay. The DPPH (2,2-diphenyl-1-picrylhydrazyl), the ABTS + (2,2′-Azino-bis 3-ethylbenzothiazoline-6-sulfonic acid di-ammonium radical salt cation), and Trolox (6-hydroxy-2,5,7,8-tetramethylchroman-2-carboxylic acid), chemicals, solvents, and media were purchased from Sigma-Aldrich Chemie GmbH^®^, Eschenstr, Taufkirchen, Germany.

### 2.2. Organisms and Microorganisms 

Freeze-dried cysts were purchased from an aquarium store in Alexandria, Egypt. The normal human liver (THLE-2) and healthy human oral epithelial (OEC) cell lines were obtained from the Egyptian company for the production of vaccines, sera, and drugs (VACSERA, Cairo, Egypt). Applied microorganisms were classified as Gram-positive, Gram-negative, and toxigenic fungi. The Gram-positive strains included *Clostridium perfringens* ATCC 13124, *Bacillus cereus* EMCC 1080, *Staphylococcus aureus* strain ATCC 33591 (MRSA-strain), and *Enterococcus faecalis* ATCC 51299. 

Gram-negative strains included *Salmonella typhi* ATCC 15566, *Klebsiella pneumoniae* ATCC 4352, *Escherichia coli* ATCC 51659, and *Campylobacter jejuni* ATCC 33560. The above isolates were obtained from the DSMZ microbial collection (which is located in Braunschweig, Germany, and is part of the Leibniz Institute DSMZ-German Collection of Microorganisms and Cell Cultures), grown on nutrient agar slants over twenty-four hours at thirty-seven degrees Celsius, and stored in a cooler at four degrees Celsius until usage. 

The fungi of toxigenic strains utilized for the assessments were *Aspergillus parasiticus* ITEM 11, *A. niger* EMCCN 10353, *A. nomius* NRRL 13137, *Fusarium culmorum* KF181, and *F. culmorum* KF846. These fungi were obtained from Toxicology and Food Contaminants, National Research Centre, Egypt. 

### 2.3. Preparation of Propolis Extract

To evaluate their bioactive content, three types of propolis extract were prepared using water, ethanol, and isopropanol solvents. Extraction procedures were performed according to the following strategy. To create the final extract, fifty grams were extracted using five hundred milliliters of extracting solution (1:10 *w/v*). A circulation system contained a shaking incubator (HZQ-311C, BLUEPARD, Pathumthani, Thailand) where the extraction process was achieved for a 16-h incubation (at 22 ± 3 °C). The mixture was filtered using Whatman No. 2 filter paper. The solution was vacuum-evaporated in a rotary evaporator (Heidolph Instruments GmbH & Co., Germany) and then lyophilized (at −60 °C/24 h) in a Dura-Dry MP freeze drier FTS System (Colton Road, East Lyme, CT 06333, USA). The resulting powder was kept cooling in amber vials until the assessments and application.

### 2.4. Determination of Phenolic and Flavonoid Contents

Using the Folin–Ciocalteu reagent in the presence of sodium carbonate Penta-hydrate, the total phenolic content of the propolis extracts was quantitatively assessed. The reducing ability determined by the Folin–Ciocalteu technique, an assay based on electron transfer, is represented as phenolic content [29]. The result was measured at a wavelength of 517 nm against the blank solution consisting of 2.5 mL EthOH and 0.5 mL distilled water. The results were presented as milligrams of gallic acid equivalents per gram of dry weight (mg GAE/g DW) using gallic acid as a standard.

The flavonoid content of each extraction type was assessed using the techniques outlined by Shraim et al. [30]. At a wavelength of 420 nm, quercetin was used as a reference. The result represented milligrams of quercetin equivalents per gram of dry weight (mg QE/g DW).

### 2.5. Determination of the Propolis Antioxidant Potency

By dissolving 1 mL of the lyophilized extracts in a methanolic solution (0.1 mM) of the DPPH (1:1; *v/v*), the scavenging capacity of propolis extracts against the DPPH was investigated [16]. The following equation was used for calculating the reduction in DPPH-radical scavenging (at 517 nm):

(1) $$\%ScA=\frac{Ab-As}{Ab}\times 100$$

where ScA is the scavenging activity calculated as a ratio percentage.

Ab is the absorbance of the control reaction (without sample).

As is the absorbance with the test compound. 

Antioxidant activity can also be measured using an assay based on ABTS+ scavenging. For the reducing power investigation [17], absorbance was measured at 700 nm using a Shimatzo spectrophotometer; Trolox solution was used as a positive control, and deionized water was utilized as a blank. The scavenging activity was calculated using the equation (Equation (1)).

### 2.6. Determination of Propolis Phenolic Fractions

As an effective total phenolic content was recorded, the propolis (EEP) ethanol extract was chosen for the following evaluations. The compounds’ presence in EEP phenolic fraction was assessed according to the method described by Stuper-Szablewska et al. [31]. The investigation was conducted with a Waters Acquity PDA detector that was part of an Acquity H class UPLC machine (Waters, Milford, MA, USA). An Acquity UPLC^®^ BEH C18 column (100 × 2.1 mm, with 1.7 µm) made by Waters in Dublin, Ireland, was used to separate substances using chromatography. The flow rate was 0.2 mL/min, injection volume was 1 µL, and column temperature was set at 33 °C. The separation was performed with a suitable gradient of the following mobile phases: A: acetonitrile with 0.1% formic acid; B: formic acid in water at a concentration of 1% (pH = 2.0). The optimization of the gradient elution program was conducted in the following manner: a starting composition of solvent A to B at a ratio of 75:25 was used at the start of the program, which lasted for one minute. This was followed by a linear gradient of solvent A to B at a ratio of 45:55, which was maintained for 10 min, then a gradient of A: B at a ratio of 30:70 up to 20 min, followed by A: B ratio at 5: 95 up to 35 min. Subsequently, an isocratic elution with a constant ratio of solvent A and solvent B at 20:80 was implemented for up to 45 min. At =320 and 280 nm the quantities of phenolic compounds were measured, and they were recognized by comparing the retention time of the marker peak to the retention time of a standard. 

### 2.7. Standard Solution Preparation

The standard compounds Protocatechuic, caffeic acid, Syringic, and vanillic acid, Catechin, Ferulic, rutin, galangin, and Kaempferol (each at a concentration of 5 mg) were introduced into a 5 mL volumetric flask and then dissolved in methanol. The standard solution was held at a temperature of 4 °C until it was ready for use. A volume of 3 mL ethanol (95%) was introduced to each propolis sample weighing 100 mg. The sample was thereafter subjected to ultrasonic extraction at a power of 380 W and a frequency of 30 KHz for a duration of 60 min. Following this, the sample was centrifuged at a speed of 2480 g for 10 min. Then, the supernatant with a volume of 0.1 mL was acquired and then diluted to a final volume of 1 mL using ethanol. Following this, the solution underwent filtration using a 0.45 μm filter. Subsequently, the obtained solution was used for the HPLC analysis.

### 2.8. Preparation of the Propolis Nanoemulsion and Nanoparticles

The nanoemulsion solution was created using the following two steps. Firstly, a 5 g of dried chitosan was weighed, added to 200 mL of acidic distilled water (1% lactic acid), and stirred (2 h/25 °C); then sodium tri-poly-phosphate solution (0.4%, *w/v*) was added. Afterward, the formed suspension was overnight stirred (500 rpm; 22 + 3 °C). By the time it ended, a glycerol-sorbitol mixture (2:1) was added at 35% (regarding chitosan weight) and then stirred (2 h). After the time was completed, several concentrations of EEP (0 ppm, 50 ppm, 100 ppm, 150 ppm, and 200 ppm) were individually added to a part of the formed solution. Each solution part continued to be stirred (30 min) until complete harmony. 

### 2.9. Determination of Propolis Nanoemulsion Properties 

Similar techniques to those reported by Malik et al. [24] were used to assess the particle size, zeta potential, poly dispersion index (PDI), and emulsion stability of prepared nanoemulsion. These significant parameters were calculated using the equipment of Malvern (Nano-S90, Zeta sizer, Malvern Panalytical Ltd., Enigma Business Park, Grove Wood Road, Malvern, UK). The following equation was used to determine the ratio of emulsion separation:

(2) $$\%\;RES=(1-(\frac{V1-V2}{V1}\;))\times 100$$

where RES is the ratio of emulsion separation.

V1 is the total volume of nanoemulsion. 

V2 is the volume of the separate solution.

The pH, acidity (as a gram of lactic acid per liter), and viscosity (as mPa) were determined by the same methodology described by Farouk et al. [25]. The emulsion characteristics were selected to reflect the stability, efficiency, and expected biological properties [32]. Also, scanning electron microscopy (Hitachi SEM, Model S-4800, Tokyo, Japan) was utilized to examine film morphology. The samples were placed in the specimen holder using double-sided adhesive tape. After vacuum sputter coating with 10 mm of gold, the samples were scanned at 1 kV with an accelerating beam voltage [33]. 

### 2.10. Cytotoxicity Evaluation for the EEP

The cytotoxicity of the EEP was examined using two different assays, brine shrimp and cell-line of two normal strains. Brine shrimp lethality bioassay (BSLBa) is a convenient system for monitoring the biological activities of various extracts. The BSLBa was completed following the procedures described by Shehata et al. [34]. However, this assay does not provide the mechanism of toxic action; this technique is excellent for determining the relative toxicity of several extracts. The cysts were hatched in simulated seawater media [35]. Toxicity was examined by adding the EEP doses (from 1 ppm to 1000 ppm) to small vials (each contained 10 shrimp hatcheries, completed in triplicate). The dose–response curve for the applied extract concentrations was generated after 3 h of incubation. 

The second evaluation adjusted the examined concentration by two cell-line strains of normal type (OCE and HepG2) according to the methodology described before [36]. Parallel, a positive control (Cisplatin) was applied for cell toxicity. The OD values were measured at 450 nm and 565 nm wavelength for MTT and SRB assays, respectively. The cell viability was calculated using MTT and SRB assays using the following equation (Equation (3)):

(3) %CV=[(ODp−ODb)/(ODc−ODb)×100%]

where CV is cell viability. 

ODp is optical density of applied propolis extract.

ODb is optical density of the blank.

ODc is optical density of the control.

### 2.11. Assessment of the Antibacterial Activity 

A diffusion test was utilized to quantify the antibacterial activity of targeted materials, as described by Abu-Sree et al. [37]. Bacterial strains used in applications were revived from lyophilized stocks in nutrient broth. In brief, examined cultured strains (Gram-positive or Gram-negative) were plated on nutrient agar (1.31–1.74 × 10^6^ CFU/mL), plates were prepared for the treatments using well-diffusion assay (0.5 cm diameter), filled with 100 µL of the test propolis concentrations (50 ng, 100 ng, 150 ng, or 200 ng), or DEMS (100 µL) as control solution was put into diffusion-wells. Petri-dish plates were incubated (24 h/37 °C), where the inhibition zone diameter of each treatment measured using digital Caliper. The effectiveness of the solutions for inhibition was measured by the clear zone (mm) size that resulted around each diffusion well.

### 2.12. Assessment of the Antifungal Activity 

The impact of applied materials for the inhibition was recorded as a clear zone diameter (mm) using the well-diffusion assay. Fungal strains were first reactivated on CzapekDox media; then, spore suspensions of each fungal strain were prepared as previously reported [38]. The spore suspensions of fungi strains were utilized for the plate-assay inoculation. Briefly, in each Petri dish of the treatments 100 µL of spore suspension for each fungus (1.02–1.24 × 10^3^ CFU/mL) were spread. In each well (0.5 cm diameter) a quantity of 100 µL suspension of 50 ng, 100 ng, 150 ng, or 200 ng propolis concentrations individually were injected. The well that was used for the control negative was filled using DMSO solution, and the well that was used for the control positive was filled using standard antifungal (nystatin). The plates were then incubated (4 days/22 ± 1 °C). The antifungal effect was evaluated against the examined strains using Czapek-dox agar media according to the CLSI methodology [39], where the well-diffusion assay was assessed using digital caliper for the considered material against the control. The greater inhibition zone diameter reflects greater antifungal efficiency.

### 2.13. Assessment of the Anti-Mycotoxigenic Activity 

The anti-mycotoxigenic action was examined using a liquid media and followed the procedures described before [40]. In brief, conical flasks of 500 mL capacity were filled using 150 mL of the YES media. Flasks were classified into six groups (with triplicates), including the control (negative and positive). The Nystatin was applied as a standard chemical antifungal compound. Flasks were autoclaved (121 °C/20 min), cooled, then inoculated with a spore suspension of *Aspergillus parasiticus* ITEM 11 at a concentration of (1.74 × 103). Flasks were incubated (21 ± 2 °C/4 days) for mycelia growth evaluations. Mycelia growth reduction (%) was calculated by the weight of mycelia dry weight, which dried at 50 °C using a hot air oven until constant weight, compared to the control. A copy of these groups was incubated (at 28 ± 2 °C/10 days) for aflatoxin production evaluations. Aflatoxin content was determined in the filtrate media using VICAM techniques and the same apparatus as described previously [41].

For anti-*Fusarium* examination, the synthetic nutrient broth (SNB) was applied for spore inoculation of *Fusarium culmorum* KF846 according to the methodology of Moura et al. [42]. Spore inoculation was completed using a 1.56 × 10^3^ spore/mL prepared spore suspension. Flasks were classified for treatment by chitosan nanoemulsion loaded with 0, 50, 100, 150, and 200 ng propolis extract and the control (free of nanoemulsion treatment). The degradation of the mycelia growth of *Fusarium* fungi and the ability for zearalenone reduction was determined by the same methodology using the VICAM instrument (VICAM Series 4EX Fluorometer) as reported before [43]. 

### 2.14. Data Statistical Analysis

Data were represented as the mean ± standard deviation (SD) that were calculated of measurements conducted in triplicate. The data were analyzed using the SPSS (V.16) statistical software. Duncan’s multiple range test and an analysis of variance (ANOVA) were utilized to determine the statistically significant difference between the means (*p* = 0.05).

## 3. Results

### 3.1. Total Phenolic and Flavonoid of Propolis Extracts

**Figure 1:**
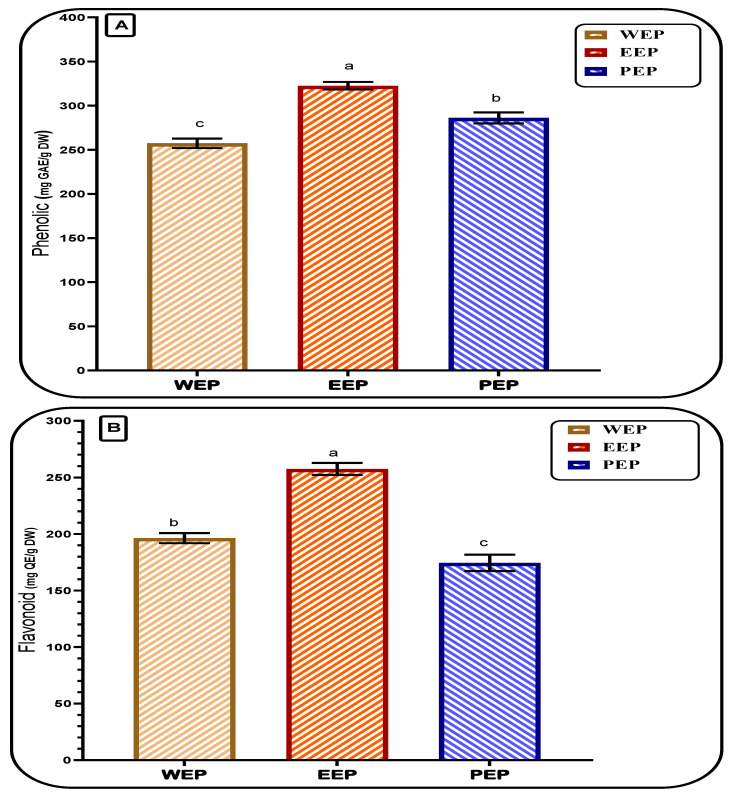
Total phenolic and total flavonoid contents of different propolis extracts. WEP: propolis water extract; EEP: propolis ethanol extract; PEP: propolis propanol extract. For each graph, the different letters reflect a significant difference between extraction methods. (**A**) represents the total phenolic of propolis extracts. (**B**) represents the total flavonoid of propolis extracts.

The total phenolic content of the gained EEP reflected a significant content according to the Folin–Ciocalteu reagent procedures (Figure 1). Among three types of propolis extraction (water, ethanol, and propanol), results showed a high content of the total phenolic in the ethanol extract. A significant change for the propolis content of phenolic and flavonoid content was noticed by the change of the solvent extraction. The flavonoid content using ethanolic extraction was the highest value, followed by the water extraction, and isopropyl extraction came last. Otherwise, for the extraction content of total phenolic, ethanolic extraction was the best, followed by isopropyl extraction, and the water extraction content of total phenolic was the lowest among the applied methods. The value recorded was 322.57 ± 4.28 mg GAE/g of dry weight. However, the EEP content of total flavonoid was 257.64 ± 5.27 mg QE/g of dry weight. The content of the phenolic compound was arranged as EEP > PEP > WEP; this order was changed for the content of flavonoids in each extract (EEP > WEP > PEP). This point could be related to the compounds’ selectivity and solubility. 

These obtained results agreed with the previous investigation, which utilized several ethanol/water concentration systems against distilled water for the extraction [44]. The result of that study reported an increment of extraction yield by increasing the ethanol to the water ratio. Moreover, the biological activity of the extract was also enhanced. Total phenolic content is a property of the extract that is linked to its antioxidant activity [45]. It also reported changes from one area to another; moreover, it could be doubled sometimes in the same country with different regions [46]. Also, several flavonoids, including pinobanksin, pinocembrin, and kaempferol, were shown to have higher concentrations in the ethanol/water extraction system than in the WEP system [44]. The previous investigation points out the significance of flavonoids as functional components found in plant sources. Due to their unique biological features and frequency in the phenolic fraction, flavonoids are the most promising candidates for assessing propolis products’ quality. Several propolis extracts were examined, demonstrating the intense free radical scavenging activity, suggesting propolis might be a valuable source of natural antioxidants. Again, the antioxidant power of propolis extract could be boosted by the solvent type utilized in the extraction process [47]. This point is clearly shown in the result of Figure 1A,B.

These obtained results agreed with the previous investigation, which utilized several ethanol/water concentration systems against distilled water for the extraction [44]. The result of that study reported an increment of extraction yield by increasing the ethanol to water ratio. Moreover, the biological activity of the extract was also enhanced. Total phenolic content is a property of the extract that is linked to its antioxidant activity [45]. It also reported changes from one area to another; moreover, it could be doubled sometimes in the same country with different regions [46]. Also, Several flavonoids, including pinobanksin, pinocembrin, and kaempferol, were shown to have higher concentrations in the ethanol/water extraction system than in the WEP system [44]. The previous investigation points out the significance of flavonoids as functional components found in plant sources. Due to their unique biological features and frequency in the phenolic fraction, flavonoids are the most promising candidates for assessing propolis products’ quality. Several propolis extracts were examined, demonstrating the intense free radical scavenging activity, suggesting propolis might be a valuable source of natural antioxidants. Again, the antioxidant power of propolis extract could be boosted by the solvent type utilized in the extraction process [47]. This point is clearly shown in the result of Figure 1A,B.

### 3.2. Antioxidant Activity of Propolis Extracts

**Figure 2:**
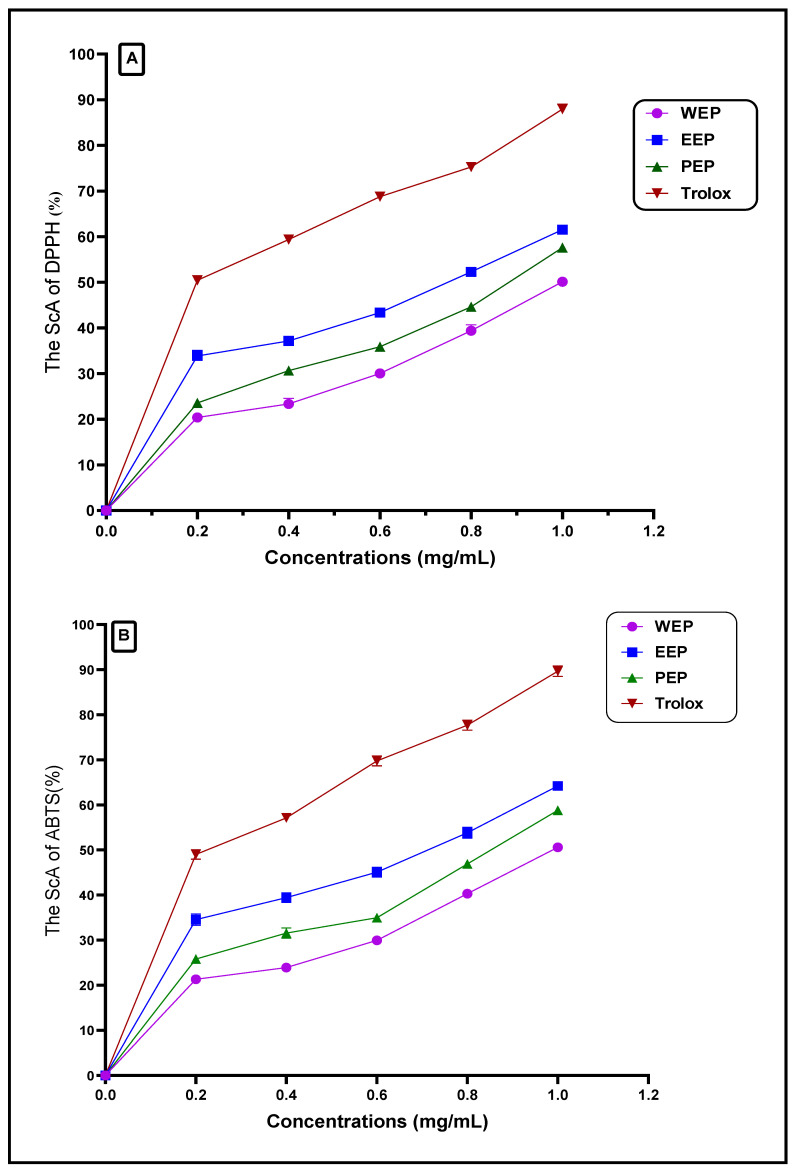
Antioxidant activity of different propolis extracts using DPPH and ABTS+ assays. WEP: propolis water extract; EEP: propolis ethanol extract; PEP: propolis propanol extract; ScA: scavenging activity. The lines in the graph for each value represent the standard deviation. (**A**) represents the DPPH-scavenging of propolis extracts. (**B**) represents the ABTS-scavenging of propolis extracts.

Evaluation of the three propolis extracts (WEP, EEP, and PEP) reflects a variation in the scavenging activity of the resulting extract (Figure 2). Examining extracts using the DPPH assay points out the EEP as the extract with the most antioxidant scavenging activity (Figure 2A). The EEP extract showed an activity close to the standard antioxidant applied as reference (Trolox). On the other hand, water extract was demonstrated by the lowest antioxidant scavenging activity. 

The evaluation of the EEP antioxidant potency was emphasized by the ABTS+ assay, where the results reflect the same by the DPPH scavenging assay; this scavenging potency was ordered as EEP > PEP > WEP in descending order (Figure 2B). Again, the results reflected significant changes for the antioxidant potency of propolis extract by the change made for solvent extraction application. The antioxidant scavenging ratio of propolis ethanolic extract (1 mg/mL concentration) is shown equal to 61.54 ± 1.05% and 64.2 ± 0.97% for DPPH and ABTS assays, respectively. The lower antioxidant scavenging ratio of propolis extract showed for the extraction process using distilled water, which are recorded at 50.11 ± 1.02% and 50.57 ± 0.67% for DPPH and ABTS assays, respectively. The values for standard antioxidant activity using Torlox at same concentration were recorded at 87.94 ± 1.10% and 89.67 ± 1.18% for DPPH and ABTS assays, respectively.

However, the antioxidant values recorded for the isopropyl extract of propolis were 57.6 ± 0.50% % and 58.81± 1.05% for DPPH and ABTS assays, respectively. The high antioxidant activity recorded for the EEP recommend it for further evaluation and more applications. This feature of antioxidant potency may have a link to the previous result of high phenolic and flavonoid content (Figure 1).

Moreover, an earlier study by Sun et al. [44] referred to the high antioxidant properties of ethanol propolis extracts that were assessed with different methods. It was pointed out that the ethanol/water systems with a greater complex phenol concentration had more substantial antioxidant efficacy than water extracts with lower phenolic content. The activity of propolis extract depends on the phenolic compound content and its types. The change in the phenolic compounds’ kind and class can affect the extract and acts as an antioxidant [48]. Again, the position of the hydroxyl group, its count, and its ability to break down an electrons-free release are linked to the antioxidant potency powerful. This potency can feature a type of propolis extract more than others for anti-illness, immune support, and biological activity.

### 3.3. Phenolic Fractions of the EEP 

The previous result of activity and content distinguished by the EEP was chosen for the next steps of evaluation and assessments. As a result, numerous phenolic and flavonoids were identified and determined in the EEP (Table 1). It was noticed that *p*-hydroxybenzoic had the higher compound content (171.75 ± 1.64 µg/g), followed by (alkaloids) caffeine (68.78 ± 1.18 µg/g). 

The content of gentisic (1.38 ± 0.05 µg/g) and vanillic (1.78 ± 0.02 µg/g) was recorded as the lowest phenolic acid contents in the EEP. Some derivatives of phenolic acid are presented such as: 3,4-dihydroxybenzaldehyde (8.96 ± 1.05 µg/g), 3,4-Dihydroxyphenol acetate (9.78 ± 0.88 µg/g), and di-methoxy cinnamic that represented the second major phenolic content (141.28 ± 1.86 µg/g). The flavonoid content is recorded by a higher content of pinocembrin (695.91 ± 1.76 µg/g), followed by quercetin (532.35 ± 1.88 µg/g), where naringin (220.96 ± 1.97 µg/g) came as the third (Table 1). The catechin flavonoid compound is recorded as the lowest flavonoid fraction content identified in the EEP, with 6.95 ± 0.03 µg/g. It was noticed that some flavonoids are represented by two derivatives such as ferulic and rutin. 

Also, functional flavonoids such as pinobanksin, galangin, apignin, and acacetin were identified by a significant content. Phenolic acids are a significant category of polyphenols that are commonly found in the human diet. Phenolic acids, easily absorbed through digestive tract walls, benefit human health by acting as antioxidants, preventing cell damage caused by free-radical oxidation processes, and promoting human anti-inflammation potential [49]. Various fruits, vegetables, and plants contain flavonoids, a class of phenolic compounds. Numerous studies have shown their positive effects on health, particularly in lowering the risk of developing serious illnesses like cancer and heart disease [50]. 

Propolis from specific regions has been shown to have better phenolic compounds than others. Phenolic acids are a smaller group of phenolic compounds in many plant-based foods. Also, it has been reported to possess anti-inflammatory and antioxidant activity and is a potential application against illness cases [49]. Studies have examined propolis’s ability to support wound healing, strengthen the immune system, and potentially prevent cancer. Antioxidant substances such as flavonoids, phenolic acids, and terpenoids have been identified in propolis extract [51]. These substances can potentially decrease oxidative stress and cell damage by destroying free radicals in the body [52]. Propolis extract was effective against breast, colon, and prostate cancer [53]. Inflammation is an essential contributing factor to many chronic illnesses, and a different study found that propolis may help lower the probability of these illnesses [54].

### 3.4. Characterization of Propolis Nanoemulsion 

The formed emulsion of chitosan, which contained several concentrations of propolis extract as EEP (50, 100, 150, and 200 ng), was assessed for its six characteristics (Table 2). Firstly, the particle size was recorded as more minor for the emulsion loaded with 50 ng of the EEP, followed by the emulsion loaded with 150 ng of the EEP. However, a more significant size was determined for the emulsion loaded by 100 ng of the EEP. This feature may affect the emulsion penetration and its biological activity [55]. Formed emulsions were examined for their Zeta potential, which concerns the emulsion stability. 

The emulsion recorded by a high charge negative value was the one loaded by 150 ng EEP, while the lowest emulsion in Zeta value was joined to the one loaded by 200 ng EEP. Regarding the Zeta values, the result has no significant differences for the emulsion loaded by 50 ng, 100 ng, and 200 ng of EEP. The polydispersity values of the emulsions with several concentrations were recorded by low value for the 150 ng-EEP loaded emulsion, which indicates its stability. However, no significant differences in the DPI values were recorded between 50 ng and 100 ng-EEP loaded emulsion. The viscosity was recorded as the highest for the 200 ng-EEP loaded emulsion, followed by the 150 ng-EEP loaded emulsion, where 50 ng-EEP loaded and 100 ng-EEP loaded were recorded by non-significant differences. The pH values and acidity were noticed to increase with the increment of the EEP concentration loading.

### 3.5. Cytotoxicity Assessment 

**Figure 3:**
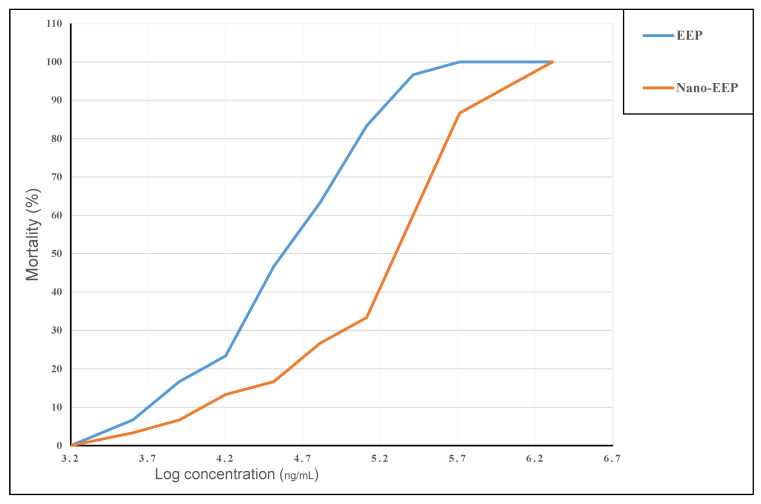
Cytotoxicity assessment of the EEP and nano-EEP brine shrimp lethality bioassay.

The cytotoxicity of raw and nanoemulsion concentrations of the EEP was determined against the brine shrimp larva assay (Figure 3). The results referred to concentrations of 2 mg/mL of the EEP added to the artificial seawater media with brine shrimp larvae as a cytotoxic dose. The results in Figure 3 indicate a lower IC50 value of EEP-nanoemulsion than the same concentration applied as a raw extract.

The IC_50_ value of the raw extract was recorded at 30 µg/mL, while this value reached 200 µg/mL for the nanoemulsion form. Moreover, the cell line toxicity determined for the EEP (raw and nanoemulsion) is recorded in Table 3. The results referred to the IC50 value for the EEP at 218.51 ± 2.08 and 237.18 ± 1.17 μg/mL for the MTT and SRB assays, respectively, on the oral epithelial cell line. These IC50 values were recorded at 227.16 ± 1.81 and 249.27 ± 1.44 μg/mL for the MTT and SRB assays for the liver THL-2 cell line. These values were higher than the toxicity doses recorded by the standard Cisplatin compound for the two types of cell lines. Regarding the IC50 values of the EEP nanoemulsion, it was recorded at 408.21 ± 1.02 and 381.16 ± 1.31 μg/mL, respectively, for MTT and SRB assays (on OEC cell line), while they were recorded at 349.66 ± 1.41 and 337.63 ± 1.18 μg/mL for MTT and SRB assays (using THL-2 cell line), respectively.

### 3.6. Antibacterial Assessment of the EEP

The EEP antibacterial activity recorded in Table 4 is assessed against pathogenic strains of Gram-positive and Gram-negative bacteria. At several concentrations of the EEP (loaded or non-loaded on chitosan), the result reflected antibacterial efficiency, which rises with the elevation of the concentration applied. Moreover, the efficiency of chitosan nanoemulsion loaded by the EEP was significant compared to the raw extract application against bacteria. More antibacterial efficiency is recorded for the Gram-positive strains than the Gram-negative ones. This phenomenon could be linked to the bacterial cell wall membrane structure and its components. 

When evaluating the activity of propolis extract against pathogenic bacteria in Table 4, the results showed no significant differences between the crude and the nanoform extracts. At the same time, significant differences were recorded as clear at high concentrations (150 and 200 ng propolis). The free-film of chitosan used for propolis loading showed limited activity compared to that loaded with propolis. The results recorded for inhibiting bacteria using a nano-propolis solution at a concentration of 200 g were so close to those recorded for applied standard antibiotics. In contrast, the inhibition results using 150 ng propolis extract in nanoform solution were slightly less than them, considering that inhibition is still considerable.

The antibacterial impact against Gram-negative bacteria using loaded EEP-chitosan showed a more significant difference than the EEP itself. This result could point out the enhancement that occurred for extract efficiency. Also, that could be linked to the size and distribution of the EEP of loaded chitosan emulsion [41]. Generally, several types of propolis were known to possess antibacterial activity [18,56]. Previous investigations also referred to the significant antibacterial activity of propolis from different regions against the Gram-positive bacteria [57]. Propolis collected from different regions of Turkish areas showed variable antibacterial activities [58]. Basically, these investigations were referred to the propolis incorporation with other substrates to enhance these antibacterial properties [59]. Also, the activity was linked to the propolis content of bioactive components such as phenolic, flavonoids, and antioxidants. Phenolic compounds from propolis or its fortification are crucial in supporting propolis antibacterial [45,48]. It has been hypothesized that propolis’s phenolic and flavonoid components are responsible for its antibacterial effects. 

Several molecules identified in propolis extracts have been demonstrated to work together to damage cell membranes; these include galangin, pinocembrin, and chrysin, of the phenolic compounds [56]. The phenolic fraction of the present propolis type reflects its rich content of these flavonoids, which could explain the distinguished antibacterial properties against the applied pathogenic strains (Table 4). The results also referred to antibacterial of the applied extracts against the MRSA strain, which is considered a great issue of bacterial contamination. This result may suggest more investigation regarding this strain in vivo and biological activity in the presence of propolis extracts.

### 3.7. Antifungal Assessment of the EEP

The antifungal effect of the EEP against toxigenic strains of fungi using loaded EEP-chitosan was more significantly different compared to the EEP itself. This result could indicate the ameliorative effect of the chitosan loading process (Table 5). For each fungus strain, the efficiency of nano-EEF emulsion showed more inhibition than the application of the EEP raw extract. Moreover, the antifungal effect showed more efficiency in *Fusarium* fungi strains compared to the Aspergilli. This result may be linked to the strain sensitivity of each fungi family. It was noticed that, for some treatments, no significant differences were recorded between the application of loaded chitosan of 150 ng and 200 ng EEP. Again, the inhibition effect is shown close to the standard antifungal at these concentrations of nanoemulsion, particularly for the *Fusarium* fungi. 

Otherwise, it was recorded that the antimicrobial impact could be enhanced by the differentiation of polyphenolic content, types, their derivatives, and antioxidant activity [45]. The antifungal activity of propolis is mainly joined to its content of flavonoids [56], which could functionally play as scavenging for the free radical [17,60]. Also, propolis phenolics’ unique content nominates it for antifungal application in alternative and complementary medicine [56].

### 3.8. Assessment of Nanoemulsion Anti-Mycotoxigenic Activity 

The nanoemulsion with several concentrations was applied in synthetic liquid media containing spore suspensions individually of Aspergilli or *Fusarium* strains. The results in Table 6 showed effective inhibition of fungal mycelia growth, particularly for the *Fusarium* fungi strain. It was noticed that 200 ng of chitosan-loaded EEP completely inhibits *Fusarium*’s fungi growth and suppresses the zearalenone production in media. Otherwise, the concentration of 150 ng chitosan-loaded EEP can only suppress zearalenone formation in the liquid media of fungal growth, but the inhibition ratio of fungal growth was recorded at 78.31% ± 0.299. For the concentrations of 50 ng and 100 ng chitosan-loaded EEP, the inhibition ratio is 19.63% ± 0.306 and 48.13% ± 0.265, respectively.

Evaluating the antifungal activity concerning the applied concentrations of propolis (50, 100, 150, 200 ng) reflects their effectiveness in fungal contamination reduction, whether for fungal growth or their metabolites (mycotoxins). The results showed the significance of propolis at the applied concentrations in decreasing the growth of both *Aspergillus* and *Fusarium* fungi (Table 5). With the increment in the used concentration of propolis, the inhibition rate increased for both the *Aspergillus* and the *Fusarium* fungi (noting that the rate of inhibition was recorded to a greater degree for *Fusarium*). At a 200 ng propolis extract concentration, the inhibition rate was 47.18 ± 0.264% for the *Aspergillus* fungi, while no growth was recorded for *Fusarium* fungi in their media. On the other hand, the results showed a significant decrease in the concentrations of aflatoxins (AFB_1_, AFB_2_, AFG_1_, and AFG_2_) with an increment in the applied propolis extract concentration in fungal growth media. Still, the results did not indicate that the fungi stopped producing aflatoxins in their growth media. Regarding the *Fusarium* fungi, the results reflected a high ability of both concentrations (150 and 200 ng propolis extract) to inhibit its mycelia growth at rates of 78.31 ± 0.299% and 100%, respectively. Again, no zearalenone concentrations were recorded in *Fusarium* growth media at those concentrations of the nano-propolis extract (150 and 200 ng). The maximum inhibition ratio achieved by applying chitosan-loaded EEP (50, 100, 150, and 200 ng/mL) was recorded at 47.18% ± 0.264, which was recorded for 200 ng-EEP-loaded emulsion. Regarding the applied concentration, 47.18% inhibition of *A. parasiticus* is the highest inhibition recorded by chitosan-loaded EEP. In further investigations, the higher concentration could also be evaluated, against several *Aspergilli* strains that produce aflatoxins. It is essential to point out the significance of Table 5 results for several applications, including pharmaceutical, nutraceutical, and food processing.

## 4. Conclusions

Enhancing the biological characteristics of ethanol propolis extract is a promising step to valorize its biological activity. EEP’s total phenolic, flavonoid, and antioxidant activity has better properties compared to the prepared extracts of PEP and WEP. The EEP analysis for phenolic fractions reflected its content of Phenolic fraction and is distinguished by 18 phenolic acids and 12 flavonoid compounds. The majority content of phenolic acids in ethanolic extract of propolis was recorded for Pinobanksin (187.72 ± 1.59 µg/g), followed by *p*-hydroxybenzoic (171.75 ± 1.64 µg/g). However, content of gentisic (1.38 ± 0.05 µg/g) and vanillic (1.78 ± 0.02 µg/g) was recorded as the lowest phenolic acid. Regarding alkaloids, it was also represented by significant content such as caffeine and caffeic acid. Flavonoid fractions of propolis extract were featured by pinocembrin (695.91 ± 1.76 µg/g), and quercetin (532.35 ± 1.88 µg/g).

The EEP was also distinguished by phenolic acid derivatives (3,4-dihydroxy benzaldehyde, 3,4-Dihydroxyphenol acetate, and di-methoxy cinnamic). The EEP cytotoxicity examined using BSLBs assay reflects that the IC50 values of the raw extract and nanoemulsion were 30 µg/mL and 200 µg/mL, respectively. However, these values were recorded by greater values using a cell line assay. Significant antibacterial activity against pathogenic bacteria, especially *Staphylococcus* (MRSA-*Staphylococcus aureus* ATCC 55391), and toxigenic fungi, especially *Fusarium*, was seen when EEP was loaded on chitosan nanoemulsion at 50, 100, 150, and 200 ng concentrations. The nanoemulsion loaded with 150 ng of EEP was the most effective of the four tested concentrations. Zearalenone synthesis in a simulated medium from *Fusarium* is completely inhibited at EEP doses of 150 and 200 ng in chitosan nanoemulsion. It inhibited fungi growth (by as much as 47.18%) and decreased aflatoxin generation in conditions containing *Aspergillus*. These findings supported using EEP-chitosan in medical facilities, pharmacological applications, and food safety scenarios.

## Figures and Tables

**Table 1:** Phenolic and flavonoid fractions of ethanol-extract for Korean propolis.

Phenolic Acids			
Compound	(µg/g)	Compound	(µg/g)
Gallic	11.68 ± 1.21	3,4-Dihydroxybenzaldehyde	8.96 ± 1.05
Protocatechuic	14.02 ± 1.37	3,4-Dihydroxyphenol acetate	9.78 ± 0.88
*p*-hydroxybenzoic	171.75 ± 1.64	Syringic	15.35 ± 0.74
Gentisic	1.38 ± 0.05	Vanillic	1.78 ± 0.02
Iso-chlorogenic	4.79 ± 0.08	Sinapic	4.85 ± 0.05
Chlorogenic	2.76 ± 0.05	*p*-coumaric	4.32 ± 0.08
Caffeic	68.78 ± 1.18	Rosmarinic	23.54 ± 0.81
caffeine	158.14 ± 1.89	Di-methoxy cinnamic	141.28 ± 1.86
Flavonoids			
Compound	(µg/g)	Compound	(µg/g)
Catechin	6.95 ± 0.03	Rutin-hydrate	47.88 ± 0.97
naringin	220.96 ± 1.97	Quercetin	532.35 ± 1.88
Ferulic acid	48.61 ± 1.28	Kaempferol	84.23 ± 1.05
Iso-ferulic acid	29.14 ± 1.08	Pinocembrin	695.91 ± 1.76
Apignin	165.48 ± 1.81	Galangin	89.09 ± 1.02
Rutin	53.51 ± 1.05	Acacetin	128.67 ± 1.47
Pinobanksin	187.72 ± 1.59	Chrysin	50.21 ± 1.41

Each compound concentration was expressed as mean ± SD (where n = 3, SD: standard deviation).

**Table 2:** Physiochemical properties of chitosan nanoemulsion loaded by several concentrations of propolis.

Propolis Concentration (ng/mL Emulsion)	50 ng	100 ng	150 ng	200 ng
Particle size (nm)	71.16 ± 5.21 ^a^	112.34 ± 7.36 ^d^	96.37 ± 4.31 ^b^	108.67 ± 3.55 ^c^
Zeta potential (mV)	−25.41 ± 1.11 ^a^	−24.34 ± 0.87 ^a^	−27.87 ± 0.62 ^b^	−23.89 ± 1.05 ^a^
PDI value	0.31 ± 0.02 ^b^	0.34 ± 0.01 ^b^	0.26 ± 0.02 ^a^	0.39 ± 0.03 ^c^
Viscosity (mPa/s)	1255.9 ± 7.28 ^a^	1259.4 ± 4.26 ^a^	1274.4 ± 5.71 ^b^	1308.2 ± 6.88 ^c^
pH value	6.19 ± 0.11 ^a^	6.09 ± 0.06 ^a^	5.81 ± 0.10 ^b^	5.56 ± 0.08 ^c^
Acidity (g. lactic /L)	1.71 ± 0.07 ^a^	1.78 ± 0.06 ^a,b^	1.89 ± 0.11 ^b^	1.91 ± 0.14 ^b^

Results are expressed as mean ± SD (where n = 3, SD: standard deviation). The values in each row that have different superscript letters were significantly different.

**Table 3:** Assessment of the cytotoxicity of propolis extract and nanoemulsion against HepG2 OEC and THL-2 cell lines (MTT and SRB assays).

Cell Line	Cisplatin	EEP	Nano-EEP
IC_50_ determined by MTT assay (μg/mL)			
OEC	61.47 ± 1.05 ^a^	218.51 ± 2.08 ^b^	408.21 ± 1.02 ^c^
THL-2	73.54 ± 0.84 ^a^	227.16 ± 1.81 ^b^	349.66 ± 1.41 ^c^
IC_50_ determined by SRB assay (μg/mL)			
OEC	63.04 ± 1.87 ^a^	237.18 ± 1.17 ^b^	381.16 ± 1.31 ^c^
THL-2	75.21 ± 0.46 ^a^	249.27 ± 1.44 ^b^	337.63 ± 1.18 ^c^

Each value was represented as mean ± SD (n = 3, SD: standard deviation). The LSD value of the MTT test was (3.741), and the regression coefficient value (R2) was 0.997. The LSD value of the SRB test was (2.984), and the regression coefficient value (R2) was 0.994. The data in each raw, with different superscription letters are significantly different.

**Table 4:** Assessment of antibacterial activity of chitosan nanoemulsion loaded by several propolis extract concentrations.

Bacterial/KPE	Raw Extract Evaluations						
		50 ng	100 ng	150 ng	200 ng	CF	ST-Antibio
Gram-positive				Inhibition (mm)			
Clostridium perfringens ATCC 13124	Raw	4.71 ± 1.06 a	9.08 ± 1.02 b	13.05 ± 1.12 c	17.91 ± 1.05 e	2.51 ± 1.08 g	38.61 ± 0.37 h
	Nano	4.96 ± 1.11 a	10.81 ± 0.81 b	27.71 ± 0.88 d	28.24 ± 0.97 f		
Bacillus cereus EMCC 1080	Raw	2.98 ± 1.21 a	8.71 ± 0.88 b	11.56 ± 1.24 c	15.41 ± 0.88 d	3.27 ± 0.89 g	39.55 ± 0.61 h
	Nano	4.26 ± 1.37 a	10.87 ± 0.44 c	27.01 ± 0.74 e	28.77 ± 0.46 f		
MRSA-Staphylococcus aureus ATCC 33591	Raw	3.08 ± 0.98 a	8.54 ± 0.37 c	11.21 ± 0.87 d	16.05 ± 0.74 e	3.54 ± 0.77 g	38.94 ± 0.54 h
	Nano	5.12 ± 0.54 b	11.02 ± 0.21 d	31.05 ± 0.84 f	31.38 ± 0.96 f		
Enterococcus faecalis ATCC 51299	Raw	2.83 ± 1.12 a	9.12 ± 0.69 b	13.96 ± 1.27 c	16.77 ± 0.49 d	2.89 ± 0.67 f	41.24 ± 0.59 g
	Nano	3.16 ± 0.98 a	12.19 ± 1.11 c	29.41 ± 0.64 e	30.11 ± 1.02 e		
Gram-negative				Inhibition (mm)			
Salmonella typhi ATCC 15566	Raw	1.71 ± 0.65 a	6.64 ± 1.02 c	11.95 ± 1.31 e	14.69 ± 1.02 f	1.81 ± 0.28 h	34.91 ± 0.59 i
	Nano	2.38 ± 0.21 b	8.05 ± 0.93 d	25.01 ± 0.81 g	25.62 ± 0.66 g		
Klebsiella pneumoniae ATCC 4352	Raw	2.21 ± 0.58 a	7.05 ± 0.94 b	9.68 ± 0.97 c	12.04 ± 1.15 d	1.47 ± 0.46 f	35.22 ± 0.41 g
	Nano	3.02 ± 0.27 a	11.21 ± 0.81 c	24.28 ± 0.54 e	25.05 ± 1.12 e		
Escherichia coli ATCC 51659	Raw	1.78 ± 0.69 a	7.14 ± 1.11 b	8.74 ± 1.48 b	12.78 ± 0.67 d	1.33 ± 0.51 f	35.67 ± 0.74 g
	Nano	2.38 ± 0.41 a	10.26 ± 0.66 c	23.05 ± 1.12 e	23.41 ± 1.05 e		
Campylobacter jejuni ATCC 33560	Raw	2.86 ± 1.34 a	7.02 ± 1.05 b	9.28 ± 1.24 c	14.82 ± 1.12 e	1.69 ± 0.46 g	38.81 ± 0.55 h
	Nano	3.26 ± 0.65 a	12.05 ± 0.79 d	26.37 ± 0.88 f	26.66 ± 0.78 f		

Each value was represented as mean ± SD (n = 3, SD: standard deviation). The inhibition zone diameter for each bacterium was measured in millimeter diameter (mm). CF: control film materials; ST- antibio: azithromycin used as a standard antibiotic; KPE: Korean propolis extract. For each bacterium, rows with different superscript letters are significantly different.

**Table 5:** Assessment of antifungal activity for chitosan nanoemulsion loaded by several propolis extract concentrations.

Bacterial/KPE		50 ng	100 ng	150 ng	200 ng	CF	ST-Antifung
*Aspergillus parasiticus* ITEM 11 (mm)	Raw	2.51 ± 1.11 ^a^	5.44 ± 0.89 ^b^	9.81 ± 1.05 ^c^	13.66 ± 1.21 ^d^	nd	25.33 ± 0.58 ^f^
	Nano	5.78 ± 1.02 ^b^	8.51 ± 1.02 ^c^	12.21 ± 1.02 ^d^	18.01 ± 1.11 ^e^		
*Aspergillus niger * EMCCN 10353 (mm)	Raw	3.18 ± 0.74 ^a^	6.08 ± 0.67 ^b^	10.02 ± 1.05 ^c^	14.79 ± 0.56 ^d^	1.02 ± 0.27 ^g^	25.05 ± 0.77 ^h^
	Nano	6.05 ± 0.88 ^b^	12.01 ± 1.08 ^c^	15.64 ± 1.01 ^e^	20.49 ± 1.21 ^f^		
*Aspergillus nomius * NRRL 13137 (mm)	Raw	3.72 ± 0.64 ^a^	7.69 ± 0.84 ^b^	10.44 ± 0.96 ^c^	15.01 ± 0.88 ^e^	2.37 ± 0.41 ^h^	26.61 ± 0.47 ^i^
	Nano	6.54 ± 1.01 ^b^	14.08 ± 1.05 ^d^	17.31 ± 1.18 ^f^	21.17 ± 1.05 ^g^		
*Fusarium culmorum* KF181 (mm)	Raw	8.07 ± 1.21 ^a^	17.47 ± 0.81 ^c^	31.44 ± 1.37 ^e^	44.781 ± 1.74 ^g^	4.26 ± 1.41 ^i^	61.08 ± 1.01 ^j^
	Nano	11.21 ± 1.08 ^b^	26.51 ± 1.11 ^d^	42.51 ± 2.31 ^f^	56.77 ± 1.37 ^h^		
*Fusarium culmorum *KF846 (mm)	Raw	8.46 ± 1.57 ^a^	18.98 ± 1.56 ^c^	37.37 ± 2.05 ^e^	49.34 ± 2.46 ^g^	5.02 ± 1.58 ^i^	76.64 ± 0.87 ^j^
	Nano	12.02 ± 1.37 ^b^	22.07 ± 1.02 ^d^	42.57 ± 2.03 ^f^	64.81 ± 1.34 ^h^		

Each value was represented as mean ± SD (n = 3, SD: standard deviation). CF: control film materials; ST-antifung: Nystatin used as standard antifungal; KPE: Korean propolis extract. For each fungus, rows with different superscript letters are significantly different. The inhibition zone diameter for each fungi growth was measured in millimeter diameter (mm).

**Table 6:** Assessment of chitosan nanoemulsion loaded by several propolis extract concentrations for anti-mycotic and anti-mycotoxigenic impact.

Anti-aflatoxigenic Effect					
Concentration (ng/mL)	0 **	50	100	150	200 *
Mycelia’s weight (g)	5.6844 ± 0.121 ^a^	4.3621 ± 0.454 ^b^	4.0211 ± 0.331 ^b^	3.5085 ± 0.218 ^c^	3.0278 ± 0.406 ^c^
Inhibition ratio (%)	--	23.26 ± 0.288	29.26 ± 0.226	38.28 ± 0.169	47.18 ± 0.264
Aflatoxin reduction					
Concentration (ng/mL)	Control	50 ng	100 ng	150 ng	200 ng
AFB_1_	271.37 ± 3.81 ^a^	237.66 ± 5.37 ^b^	198.08 ± 4.27 ^c^	167.17 ± 3.54 ^d^	124.69 ± 5.41 ^e^
AFB_2_	202.24 ± 2.54 ^a^	189.87 ± 5.11 ^b^	168.16 ± 4.05 ^c^	131.66 ± 3.81 ^d^	106.47 ± 4.68 ^e^
AFG_1_	255.18 ± 3.61 ^a^	223.61 ± 5.27 ^b^	181.56 ± 4.16 ^c^	147.26 ± 4.22 ^d^	118.56 ± 4.05 ^e^
AFG_2_	195.18 ± 3.66 ^a^	161.05 ± 4.15 ^b^	134.44 ± 3.08 ^c^	101.66 ± 3.89 ^d^	86.97 ± 5.41 ^e^
Anti-Fusarium effect					
Concentration (ng/mL)	0 **	50	100	150	200 ^*^
Mycelia’s weight (g)	5.1118 ± 0.324 ^a^	4.1085 ± 0.288 ^b^	2.6514 ± 0.205 ^c^	1.1088 ± 0.174 ^d^	Nd
Inhibition ratio (%)	--	19.63 ± 0.306	48.13 ± 0.265	78.31 ± 0.299	Nd
Zearalenone reduction					
Concentration (ng/mL)	Control	50	100	150	200
	874.56 ± 2.34 ^a^	391.27 ± 5.56 ^b^	79.88 ± 1.27 ^c^	nd	Nd

Each value was represented as mean ± SD (n = 3, SD: standard deviation). For each fungus, rows with different superscript letters are significantly different. (*) the applied concentration of ethanol propolis extract; (0 **): flasks serve as control with no propolis extract content.

## Data Availability

The data used to support the findings of this study are included in the article.
